# Status of Cardiac Autonomic Functions Among Hypertensive Children Identified by Ambulatory Blood Pressure Monitoring (ABPM): A Case-Control Study

**DOI:** 10.7759/cureus.114406

**Published:** 2026-08-12

**Authors:** Anzar Bashir Alvi, Ruchi Singh, Surjan Singh Yaduwanshi, Girish Bhatt, Sunil Chouhan, Ashok Kumar, Santosh Wakode

**Affiliations:** 1 Physiology, All India Institute of Medical Sciences, Bhopal, Bhopal, IND; 2 Pediatrics, All India Institute of Medical Sciences, Bhopal, Bhopal, IND; 3 Biochemistry, All India Institute of Medical Sciences, Bhopal, Bhopal, IND

**Keywords:** 24-hour ambulatory blood pressure monitoring, autonomic nervous system (ans), body mass index (bmi), children, primary hypertension

## Abstract

Introduction: Hypertension is no longer an adult-only disorder; it is increasingly seen in children and adolescents worldwide. Earlier, pediatric hypertension was mainly attributed to secondary causes, but recent evidence shows a rising prevalence of primary hypertension, largely driven by sedentary lifestyle, unhealthy diet, and increased screen time. Hypertension affects not only blood pressure levels but also cardiac autonomic function, disturbing the balance between sympathetic and parasympathetic activity. This study aimed to obtain objective evidence of altered autonomic function in hypertensive children.

Methods: It is a case-control study. Thirty hypertensive children diagnosed by 24-hour ambulatory blood pressure monitoring and 30 age-matched normotensive controls were included after ethical approval and informed consent/assent. Autonomic function was assessed using Ewing’s battery tests and a five-minute heart rate variability recording.

Results: Hypertensive children had significantly higher BMI (p<0.001), body weight (p<0.001), systolic blood pressure (SBP; p<0.001), and diastolic blood pressure (DBP; p<0.001) compared to controls. They also showed a higher low-frequency/high-frequency (LF/HF) ratio, mean heart rate, and stress index (p<0.001), indicating increased sympathetic activity. In contrast, mean RR interval, total heart rate variability (HRV) power, pNN50, and triangular index (p<0.001) were significantly lower, reflecting reduced parasympathetic activity. A significant positive correlation was observed between BMI and the LF/HF ratio, and linear regression showed that for every 1 kg/m² increase in BMI, the LF/HF ratio increases by approximately 6-7%, suggesting that higher BMI is associated with greater sympathetic activity in hypertensive children.

Conclusion: The findings indicate an early autonomic imbalance among pediatric hypertensives, which may contribute to future cardiovascular risks. It necessitates early screening of children as a routine for hypertension using ambulatory blood pressure monitoring.

## Introduction

Pediatric hypertension is a significant public health concern as it can lead to the development of cardiovascular diseases in adulthood [1]. Earlier, hypertension in children was uncommon and was usually linked to an underlying secondary cause [2]. However, recent evidence shows that in up to 91% of children evaluated for hypertension, no specific cause could be identified [3], indicating a growing prevalence of primary hypertension in the pediatric population. Rising sedentary lifestyles, accompanied by increased screen time, have increased the prevalence in India to around 7.6% [4]. In India, teenagers make up about one-fifth of the population [5], and the rate of high blood pressure (BP) among them ranges from around 2% to 21.5% [6]. Obese children with ambulatory blood pressure monitoring (ABPM) have shown a prevalence of ambulatory hypertension ranging from 25% to as high as 48.6%, with severe ambulatory hypertension in about 20% [7].

While hypertension in children is often associated with obesity, one potential cause of paediatric essential hypertension is autonomic dysregulation [8]. Recent evidence suggests the role of an increased central sympathetic outflow in the maintenance of hypertension in children [9]. Autonomic dysregulation can lead to chronic elevation of BP, which can cause damage to vital organs such as the heart, kidneys, and brain [1]. Yakinci et al. reported dysfunction of both sympathetic and parasympathetic components of the autonomic nervous system in children with essential hypertension compared to normotensives [8]. Urbina et al., in a small group of adolescents from the United States, showed predominance in sympathetic activity in the hypertensive group [10]. Though studies have explored the autonomic status of normotensive and prehypertensive children in India [11], none of the studies has reported the status of autonomic function in hypertensive children. Hence, the present study was conducted to assess cardiac autonomic function in hypertensive and normotensive children aged 6-18 years.

## Materials and methods

A case-control study was performed from June 2024 to December 2025 in agreement with the ethical guidelines of the Declaration of Helsinki, once it was approved by the institutional ethical committee. The Institutional Human Ethics Committee-Student Research (IHEC-SR), All India Institute of Medical Sciences (AIIMS), Bhopal, issued approval IHEC/SR/2024/107.

Participants

All participants between 6 and 18 years of age, reporting to the pediatric OPD with complaints of increasing body weight, increased appetite, headaches, chest pain, and dizziness, were informed about the study and were invited to participate. Children with high BP, when measured manually in the OPD clinics of the Pediatrics department, were advised to undergo 24-hour ABPM. Children having high BP after ABPM were diagnosed as hypertensive (according to cut-off values by the American Academy of Pediatrics (AAP), 2017) [12]. Children between 6 and 13 years having >95th percentile to <95th percentile+12 mmHg of BP and more than 13 to 18 years having 130/80 mmHg of BP were considered hypertensive (cases). Children without any underlying cause were identified to have essential hypertension, and those with secondary causes like chronic kidney disease (CKD) were excluded. Age-matched children with a normal range of office BP were enrolled as controls. Written informed consent was obtained from the parent/guardian, and verbal assent was obtained from the participants. A total of 60 participants were enrolled, 30 hypertensive and 30 normotensive.

Procedure

Measurements of height and weight were done by a wall-mounted stadiometer and a digital weighing machine, respectively. BMI was calculated and classified according to the WHO 2007 BMI-for-age reference (5-19 years) using age- and sex-specific Z-scores, categorizing participants as underweight (< -2 SD), normal (-2 to +1 SD), overweight (>+1 SD), or obese (>+2 SD) [13]. After obtaining sociodemographic data, all cases underwent ABPM, whereas heart rate variability (HRV) and autonomic function tests (AFT) were performed in both cases and controls.

Ambulatory Blood Pressure Monitoring (ABPM)

It was performed using a Spacelab-9022 (Spacelabs, Inc., Chatsworth, CA, USA) device. Participants underwent 24-hour ABPM with an appropriately sized cuff applied to the non-dominant arm, and BP was recorded every 20 min during waking hours and every 30-60 min during sleep. Parents maintained a diary documenting daily activities, sleep/wake time, and medication intake. ABPM variables were analysed as mean ambulatory systolic blood pressure (SBP) and diastolic blood pressure (DBP) over 24 hours, daytime and nighttime periods, BP load (percentage of readings above the 95th percentile), and percentage dipping.

Heart Rate Variability (HRV)

Recordings were obtained using BIOPAC Systems (Goleta, CA, USA) AcqKnowledge 5.0 software. Beat-to-beat R-R interval variability was analyzed to evaluate cardiac autonomic balance. ECG signals were recorded in the supine position for six minutes at a sampling rate of 1000 Hz. Participants were instructed to avoid food for two hours prior to testing, abstain from caffeine, nicotine, and alcohol for 12 hours, and wear loose, comfortable clothing. HRV analysis included time-domain parameters (SDNN, SDSD, RMSSD, pNN50), frequency-domain parameters (LF, HF, VLF, and LF/HF ratio), and non-linear measures, with spectral analysis performed using fast Fourier transformation.

Autonomic Function Testing (AFT)

Autonomic function was assessed using Ewing’s battery of tests for parasympathetic and sympathetic response. Parasympathetic activity was evaluated through heart rate responses to the deep breathing test (E/I ratio), head-up tilt test (30:15 ratio), and the Valsalva maneuver. Sympathetic activity was assessed by measuring BP responses to postural change (head-up tilt test on tilt table at 60°) and during the sustained handgrip test (HGT). Participants were grouped according to the autonomic involvement; those with all test results within the normal range were classified as having no cardiovascular autonomic neuropathy (CAN). Individuals showing early signs of autonomic dysfunction, either one abnormal heart rate test or two borderline results, were categorized as early CAN [14]. Participants with more advanced involvement, indicated by two abnormal tests and/or the presence of orthostatic hypotension, were classified as having definite CAN [15].

Statistical analysis

Data were entered into Microsoft Excel (Microsoft Corporation, Redmond, WA, USA) and analyzed using IBM SPSS Statistics for Windows, version 21 (IBM Corp., Armonk, NY, USA). Results were expressed as mean±standard deviation and percentages, with corresponding p-values. Pearson’s correlation coefficient was used to assess associations between BMI and HRV parameters in hypertensive and normotensive groups. Parameters showing significant correlations were further analysed using simple linear regression. Simple linear regression was performed to assess the relationship between selected predictor and outcome variables, and the regression coefficient (β), 95% CI, R², and p-value were reported. Group comparisons were performed using the unpaired t-test. A two-tailed p-value < 0.05 was considered statistically significant.

## Results

Baseline demographic and anthropometric characteristics

**Figure 1:**
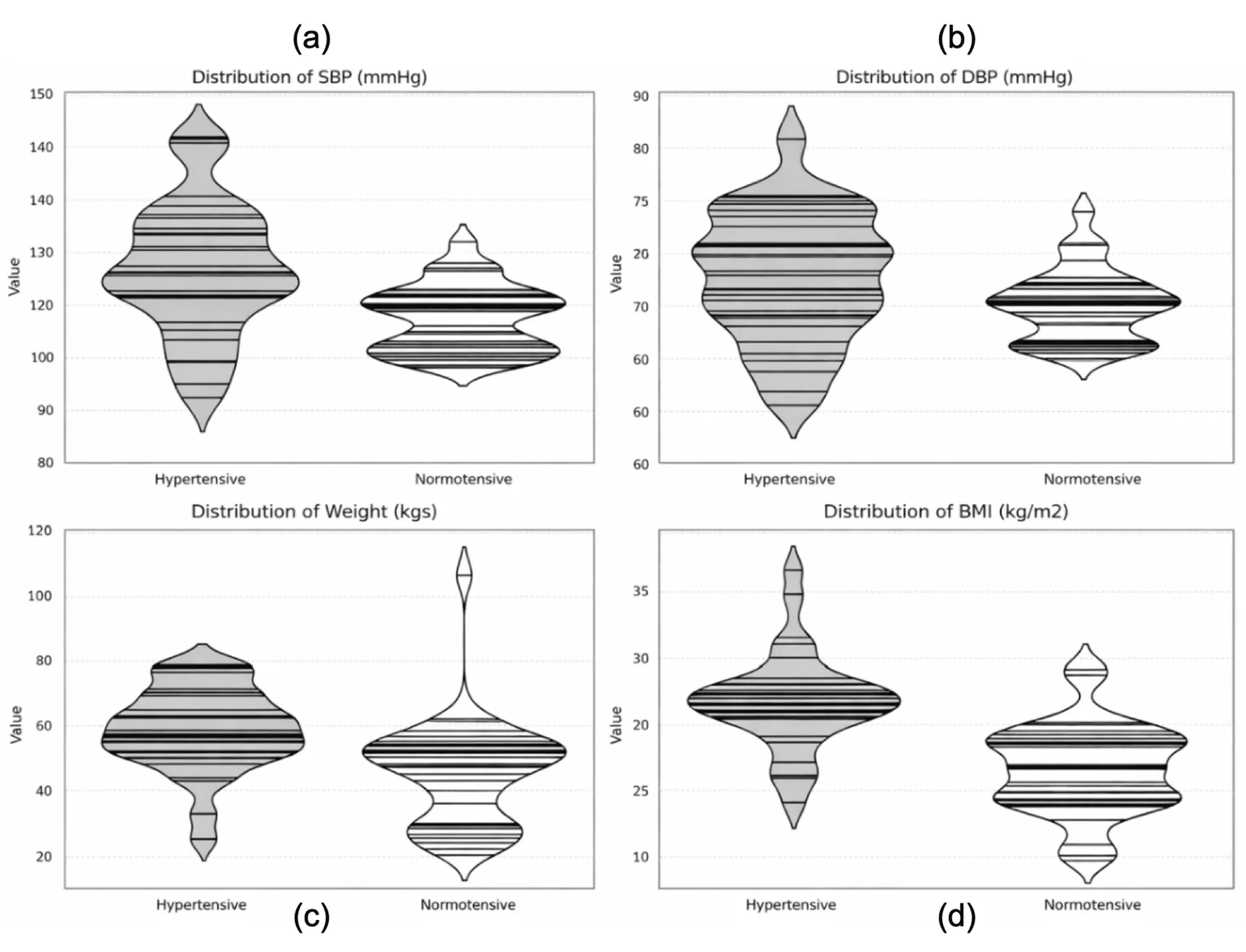
Baseline characteristics of the participants. Bean plots showing the distribution of (a) SBP, (b) DBP, (c) body weight, and (d) BMI in hypertensive and normotensive children identified by ABPM. The data represent 60 participants (biological replicates; hypertensive n = 30, normotensive n = 30) with one observation per participant and no technical replicates. SBP, systolic blood pressure; DBP, diastolic blood pressure; BMI, body mass index; ABPM, ambulatory blood pressure monitoring.

Thirty hypertensive and 30 normotensive children with a mean age of 13.21±2.43 years and 14.03±2.43 years participated in this study. The hypertensive group comprised a higher proportion of males, 23 (76.66%), compared to the normotensive group, 17 (56.66%). Office BP was significantly higher in hypertensives (SBP: 118.17 ± 15.10 mmHg; DBP: 73.90 ± 6.39 mmHg) compared to normotensives (SBP: 106.17 ± 6.61 mmHg; DBP: 69.67 ± 3.56 mmHg; p = 0.0002 and 0.0024, respectively). The height of participants was comparable (p = 0.964), but body weight and BMI were significantly higher in hypertensive participants (p < 0.001). BMI categorisation showed that most hypertensive participants were either obese (n=14, 46.7%) or overweight (n=8, 26.7%), while normotensive participants were predominantly of normal weight (n=23, 76.7%), indicating a strong association between elevated blood pressure and increased body weight (Figure 1).

**Figure 2:**
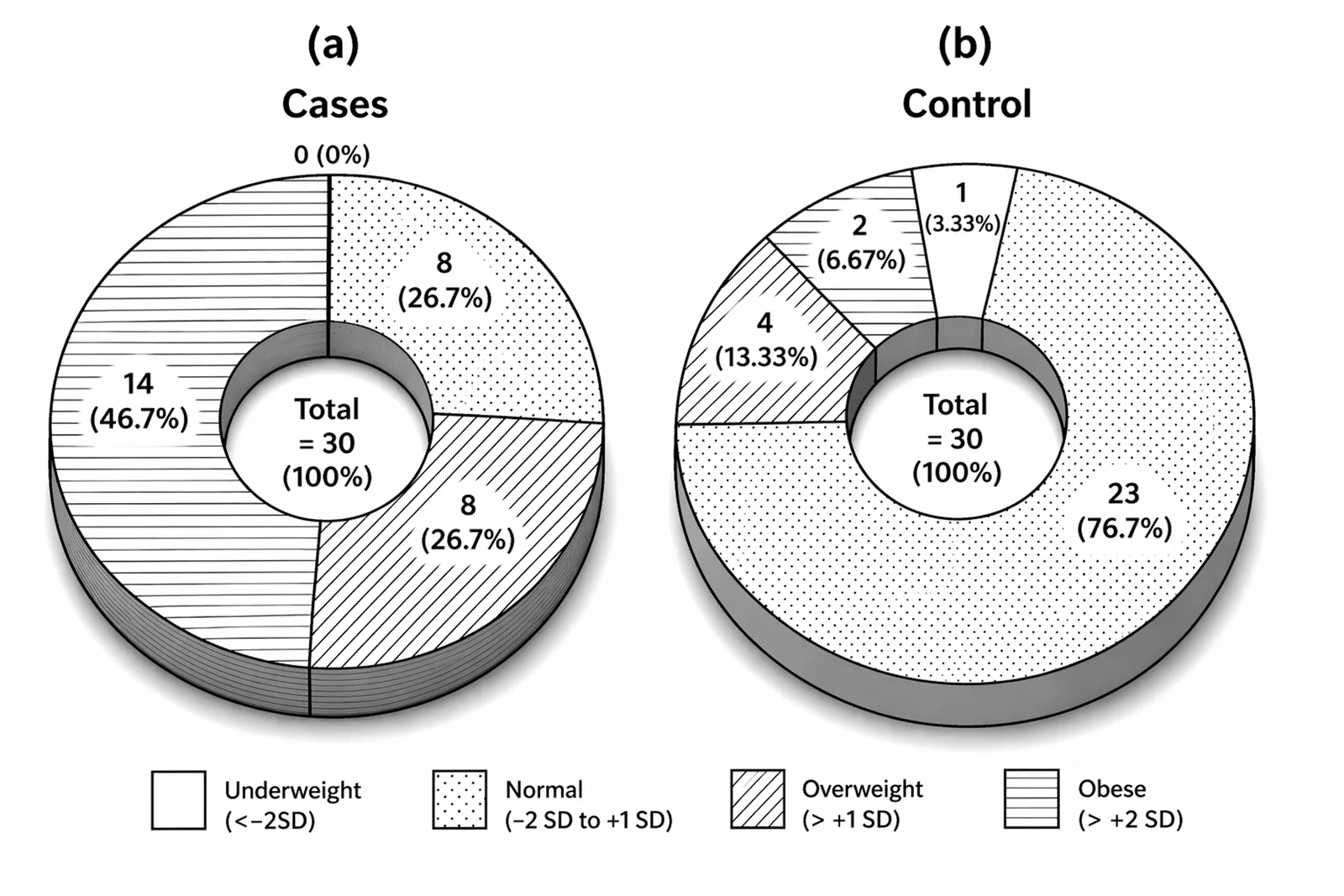
BMI distribution according to WHO (2007) classification. Doughnut charts showing BMI categories among (a) hypertensive (n = 30) children, showing a higher proportion of obese individuals, with equal distribution of normal and overweight categories, and (b) normotensive (n = 30) children predominantly within the normal BMI range based on the WHO 2007 [13] growth reference (SD scores). Categories include underweight (<−2 SD), normal (−2 SD to +1 SD), overweight (>+1 SD), and obese (>+2 SD).

Further, the participants were classified into BMI categories (Figure 2) according to WHO (2007) [13].

Blood pressure of hypertensives in office, awake, and sleep states

**Figure 3:**
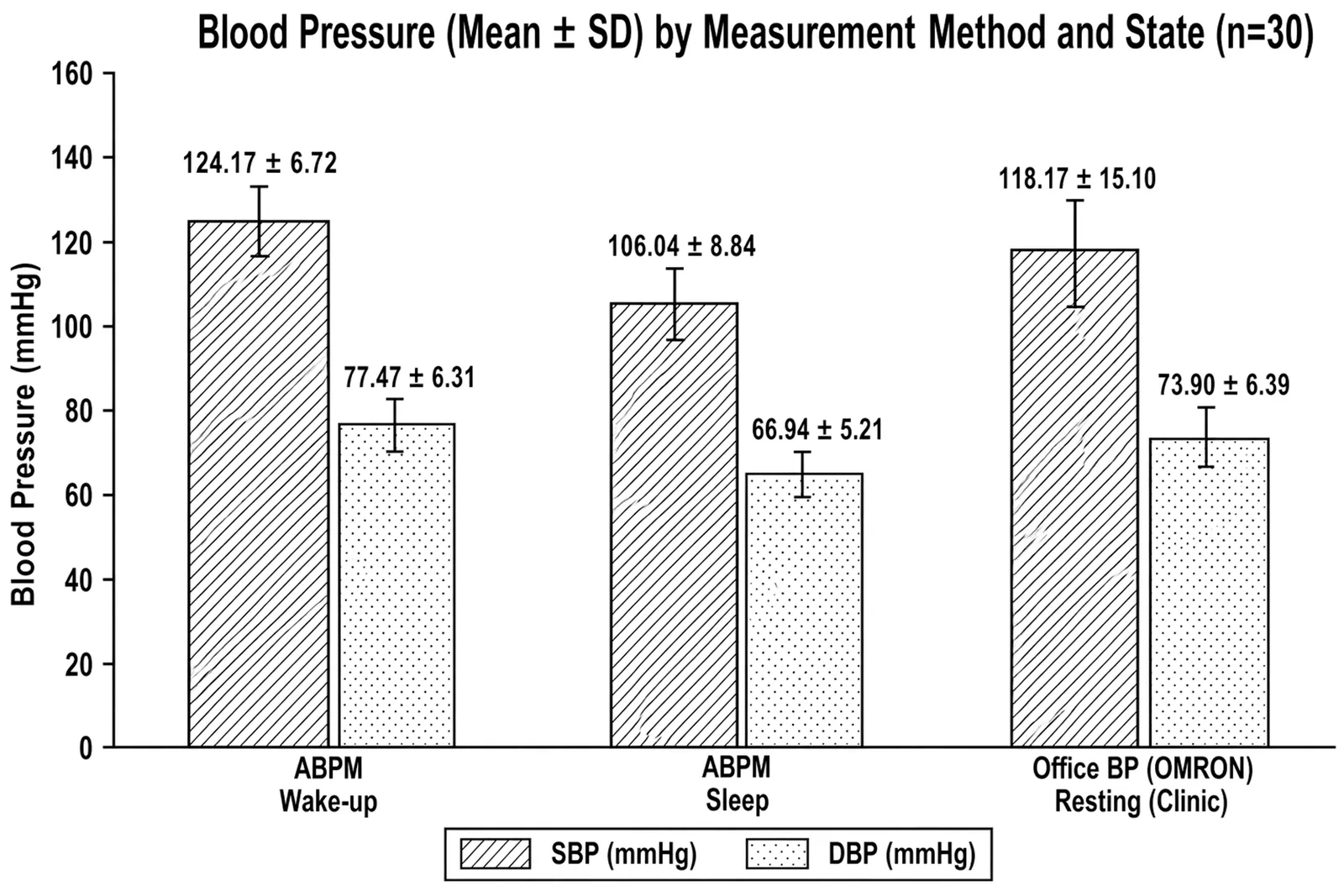
Blood pressure of hypertensive children in different states. Bar graph showing mean ± SD systolic blood pressure (SBP) and diastolic blood pressure (DBP) of hypertensive children during wake (ABPM), sleep (ABPM), and office blood pressure (Omron HEM 7124, Omron Corporation, Kyoto, Japan) measurements. The data represent 30 hypertensive participants (biological replicates) with one observation per participant and no technical replicates. ABPM, ambulatory blood pressure monitoring.

In hypertensive children, ABPM showed blood pressure variability across different states, such as awake and sleep states. Mean blood pressure was highest during the wake-up state, with SBP at 124.17 ± 6.72 mmHg and DBP at 77.47 ± 6.31 mmHg, and lowest during the sleep state, with SBP at 106.04 ± 8.84mmHg and DBP at 66.94 ± 5.21 mmHg. (Figure 3) 

Classification and distribution of hypertension based on ABPM

**Figure 4:**
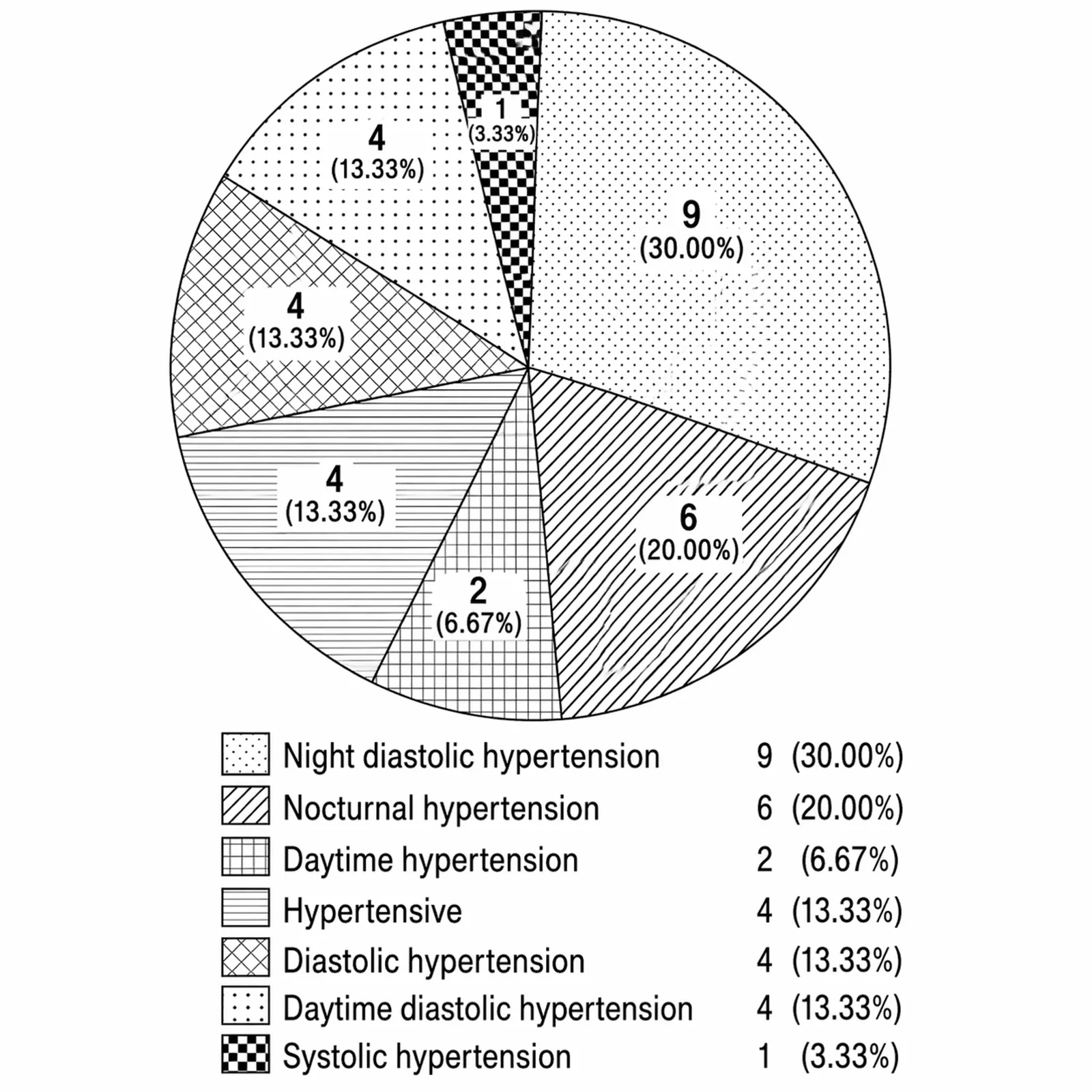
Classification of hypertension on the basis of ABPM. Pie chart illustrating the distribution of hypertension phenotypes, including systolic hypertension, diastolic hypertension, daytime hypertension, nocturnal hypertension, daytime diastolic hypertension, and night diastolic hypertension among 30 hypertensive children identified by ABPM. The data represent 30 participants (biological replicates) with one observation per participant and no technical replicates. ABPM, ambulatory blood pressure monitoring.

Night diastolic hypertension was the most common (30.00%, n = 9), followed by nocturnal hypertension (20.00%, n = 6). Hypertension, diastolic hypertension, and daytime diastolic hypertension each accounted for 13.33% (n = 4). Daytime hypertension (6.67%, n = 2) and systolic hypertension (3.33%, n = 1) were less frequent. Overall, night-time hypertension patterns were more predominant (Figure 4). Among the 30 hypertensive patients assessed by ABPM, 16 (53.3%) exhibited a dipper pattern, while 14 (46.7%) were classified as non-dippers. Chi-square analysis revealed a significant association between night-time hypertension and dipping status (OR = 0.11, p = 0.009), indicating a higher likelihood of night-time hypertension among non-dippers.

Comparison of heart rate variability parameters between hypertensive and normotensive groups

**Figure 5:**
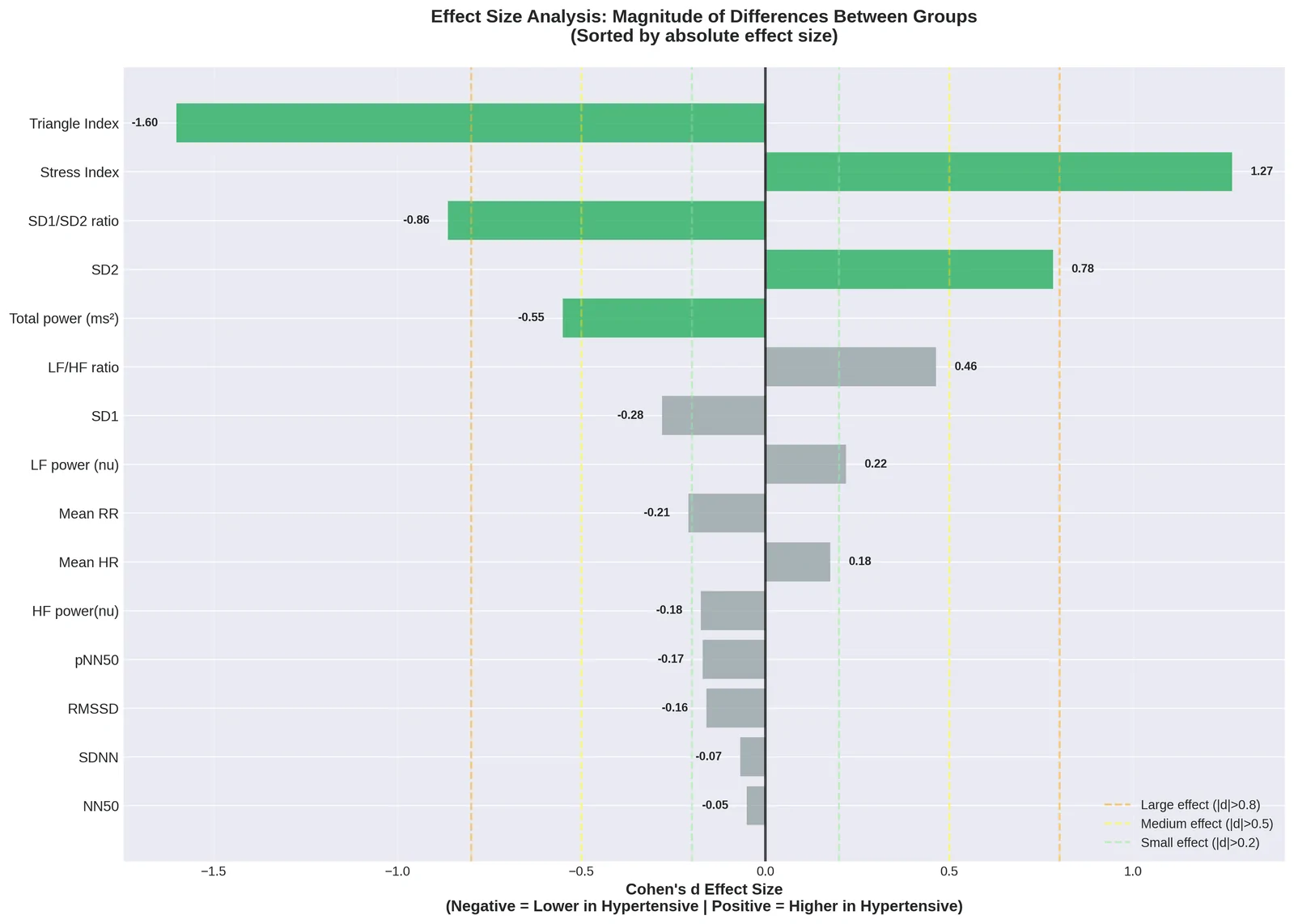
Effect size analysis of HRV parameters between cases and controls. Bar plot showing Cohen’s d effect sizes for heart rate variability (HRV) parameters comparing hypertensive cases and normotensive controls. Positive values indicate higher values in hypertensive participants, while negative values indicate lower values in hypertensive participants. The data represent 60 participants (biological replicates; hypertensive n = 30, normotensive n = 30) with one observation per participant and no technical replicates. Effect sizes are interpreted as small (|d| ≥ 0.2), medium (|d| ≥ 0.5), and large (|d| ≥ 0.8).

Heart rate variability analysis revealed no significant differences in mean RR interval, mean heart rate, or most time-domain parasympathetic indices between the hypertensive and normotensive groups. Mean RR (772.8 ± 112.26 ms, 803.0 ± 170.58 ms; p = 0.426), mean HR (79.9 ± 11.55 bpm, 77.57 ± 14.65 bpm; p = 0.495), SDNN, RMSSD, NN50, pNN50, and SD1 were comparable between groups (p > 0.05) and showed minimal between-group variation in effect size (|d| < 0.2). Effect size (Cohen’s d) analysis showed marked differences between groups, particularly for the Triangle Index (d = -1.60) and Stress Index (d = 1.27), suggesting pronounced alterations in autonomic function among hypertensive children. Moderate differences were noted in non-linear HRV measures, such as SD2 (d = 0.78) and the SD1/SD2 ratio (d = -0.86) (Table 1, Figure 5).

**Table 1:** Comparison of HRV parameters between hypertensive and normotensive children. HRV parameters measured from electrocardiographic recordings were compared between hypertensive (n = 30) and normotensive (n = 30) children. Data are presented as mean ± standard deviation (SD) and represent 60 participants (biological replicates) with one observation per participant and no technical replicates. Group differences were analysed using the independent samples t-test, and corresponding p-values are reported. HRV, heart rate variability; RR, R–R interval; HR, heart rate; SDNN, standard deviation of NN intervals; RMSSD, root mean square of successive differences; NN50, number of successive NN intervals differing by >50 ms; pNN50, percentage of NN50; LF, low frequency; HF, high frequency. * p < 0.05 was considered statistically significant.

Parameters	Physiological indication	Hypertensive (N=30)	Normotensive (N=30)	p value
Mean RR	High: Para sympathetic activity, low: Sympathetic activity	772.8±112.26	803±170.58	0.426
Mean HR	High: Sympathetic activity, low: parasympathetic activity	79.9±11.55	77.57±14.65	0.495
SDNN	Reflects overall HRV, influenced by both sympathetic and parasympathetic systems.	53.47±23.57	55.14±25.58	0.793
RMSSD	reflects parasympathetic (vagal) activity	56.44±26.84	59.82±13.09	0.537
NN50	Reflects parasympathetic activity and beat-to-beat variability	144.23±67.07	147.47±61.93	0.846
pNN50	Reflects parasympathetic (vagal) activity.	32.01±22.22	35.69±20.88	0.51
Triangle Index	It is a time-domain, reflects the overall HRV.	24.35±8.02	36.22±6.75	<0.001*
Stress Index	quantifies sympathetic activity	78.15±16.63	53.95±21.23	<0.001*
SD1	Reflects parasympathetic activity	37.45±15.82	42.19±17.85	0.28
SD2	Reflects overall autonomic balance	51.48±16.84	39.59±13.37	0.004*
SD1/SD2 ratio	Reflects overall HRV	0.73±0.21	1.17±0.69	0.001*
LF power (nu)	Mixed sympathetic–vagal modulation	50.55±15.23	47.13±15.93	0.398
HF power(nu)	Reflects paraympathetic activity	49.45±15.23	52.19 ±16.02	0.499
LF/HF ratio	indicates sympathovagal balance.	1.316±0.90	0.97±0.55	0.0767
Total power (ms2)	Reflects overall HRV	977.167±356.14	1255.67±620.37	0.037*

Frequency-domain analysis showed no significant differences in LF power, HF power, or LF/HF ratio. However, total power was significantly reduced in hypertensive participants (977.17±356.14 ms²) compared with normotensives (1255.67± 620.37 ms²; p=0.037), further supporting reduced overall autonomic variability in the hypertensive group (Table 1).

Cardiac autonomic function using Ewing’s battery

**Figure 6:**
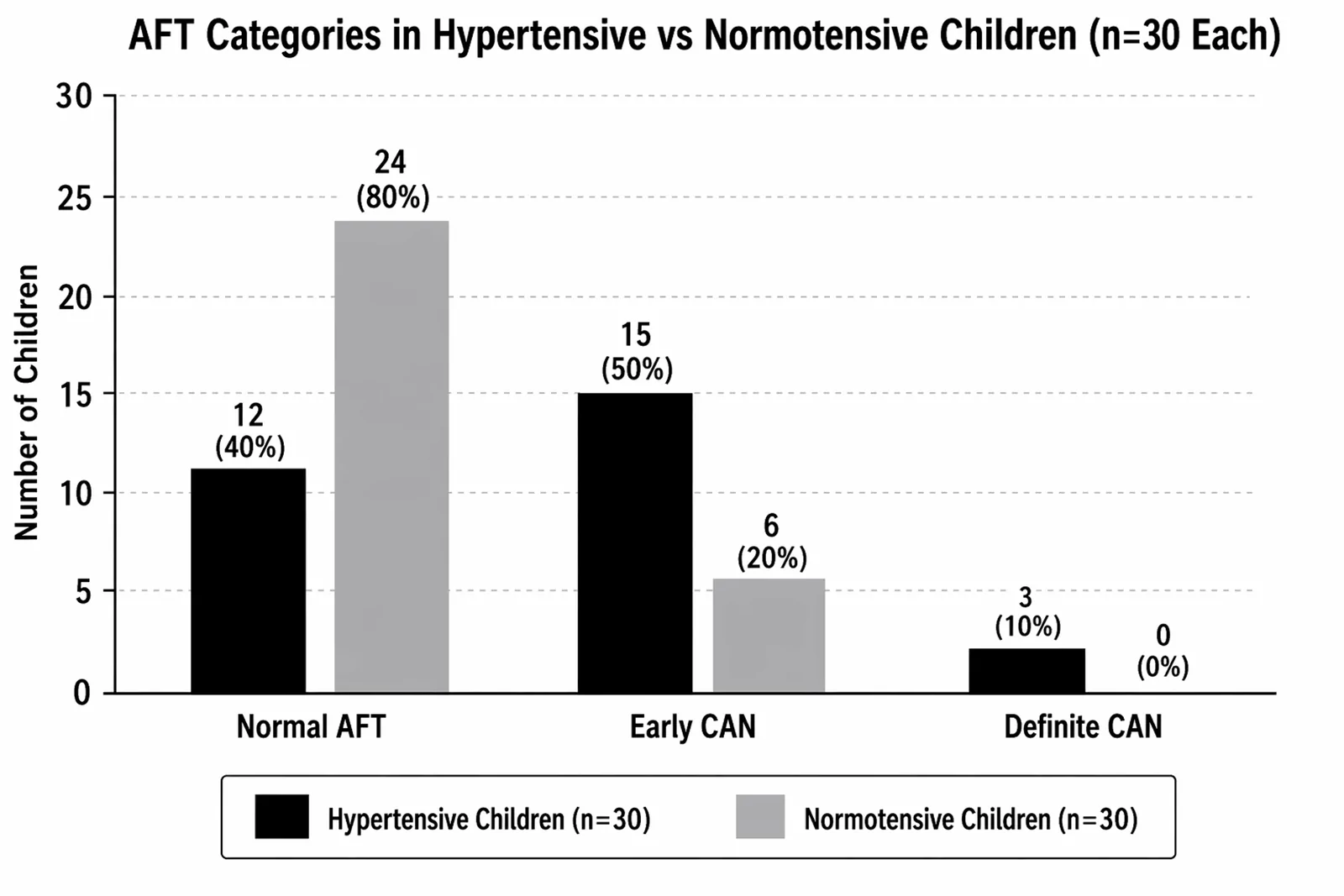
Distribution of CAN stages according to Ewing’s battery. Bar graph showing the distribution of no CAN, early CAN, and definite CAN among hypertensive and normotensive children based on Ewing’s cardiovascular tests. The data represent 60 participants (biological replicates; hypertensive n = 30, normotensive n = 30) with one observation per participant and no technical replicates. CAN, cardiac autonomic neuropathy; AFT, autonomic function tests.

Among the hypertensive children, 12 (40%) participants had normal AFT, 15 (50%) had early CAN, and 3 (10%) had definite CAN. In contrast, most normotensives showed normal AFT (n = 24, 80%), while 6 (20%) had early CAN and none had definite CAN, indicating a higher burden and severity of CAN among children with hypertension (Figure 6).

Association between body mass index and cardiac autonomic indices

In hypertensive individuals, BMI showed a significant positive correlation with the LF/HF ratio, indicating altered autonomic balance, while correlations with other HRV indices and BP were weak and non-significant. In normotensive individuals, BMI showed only weak, non-significant associations with HRV measures and BP. As the LF/HF ratio did not follow a normal distribution, a base-10 logarithmic transformation was applied to achieve approximate normality. Normality of the transformed data was confirmed using the Shapiro-Wilk test (p > 0.05). Following normalisation, Pearson’s correlation analysis was performed, and linear regression analysis was subsequently conducted to examine the relationship between BMI and the log-transformed LF/HF ratio. Linear regression analysis demonstrated that the natural log-transformed LF/HF ratio increased by 0.065 units for every 1 kg/m² increase in BMI (ŷ = 0.0648X − 1.57854), corresponding to an approximate 6-7% increase in the LF/HF ratio (Table 2).

**Table 2:** Correlation between BMI, HRV parameters, and blood pressure in hypertensive and normotensive children. Pearson’s correlation analysis showing the relationship of BMI with HRV parameters (HF power, LF power, total power, LF/HF ratio) and blood pressure (SBP, DBP) in hypertensive (n = 30) and normotensive (n = 30) children. The data represent 60 participants (biological replicates) with one observation per participant and no technical replicates. Correlation coefficient (r) and corresponding p-values were calculated using Pearson’s correlation test (two-tailed). BMI, body mass index; HRV, heart rate variability; HF, high frequency; LF, low frequency; SBP, systolic blood pressure; DBP, diastolic blood pressure. * p < 0.05 was considered statistically significant.

Variables	Hypertensive (n=30)		Normotensive (n=30)	
	r	p	r	p
BMI vs. HF power	-0.1398	0.4630	0.0406	0.8310
BMI vs. LF power	0.1398	0.4610	-0.0500	0.7930
BMI vs. total power	0.1208	0.5240	-0.0597	0.7560
BMI vs. LF/HF ratio	0.4545	0.0116*	-0.078	0.682
BMI vs. SBP	0.0579	0.7612	0.2667	0.1543
BMI vs. DBP	0.0808	0.6710	0.0229	0.9040

## Discussion

High BP in childhood and adolescence can lead to adult hypertension and cardiovascular risks, making early detection and intervention essential. In the present study, ABPM demonstrated clear state-dependent variability in hypertensive children, with mean BP values during the awake period (SBP 124.17 ± 6.72 mmHg; DBP 77.47 ± 6.31 mmHg) and sleep state (SBP 106.04 ± 8.84 mmHg; DBP 66.94 ± 5.21 mmHg), reflecting intact circadian BP rhythm. A similar trend has been reported by Lurbe et al. [16]. The American Heart Association (AHA) further emphasizes that while such diurnal variation is physiological, attenuation of the nocturnal decline is strongly associated with early target organ damage, underscoring the clinical importance of evaluating both awake and sleep BP profiles in hypertensive children [17].

In our study, nocturnal BP abnormalities predominated, with night-time diastolic hypertension (n=9, 30%) and nocturnal hypertension (n=6, 20%) occurring more frequently than isolated daytime hypertension (n=2, 6.67%), highlighting the critical role of ABPM in uncovering clinically silent nocturnal phenotypes. These findings are consistent with those of Awazu M., who reported isolated nocturnal hypertension in 16.16% of hypertensive children and in 5.55% of children without an identifiable secondary cause [18]. Importantly, Duzova et al. demonstrated that children with isolated nocturnal hypertension have a significantly higher left ventricular mass index compared with normotensives, indicating early target-organ damage [19].

Our study also shows that hypertensive children have reduced HRV, but obesity is not an essential association for hypertension. Several other factors have also been shown to contribute to arterial hypertension in children, including poor sleep, smoking, insulin resistance, increased sympathetic activity, altered sodium balance, changes in the renin-angiotensin system and endothelial function, hyperuricemia, genetic predisposition, and a history of low birth weight or prematurity in addition to obesity [20]. Perinatal stress may also contribute to autonomic nervous system dysregulation, as children born preterm or small for gestational age show higher heart rates and increased catecholamine excretion, indicating sympathetic overactivity. This early autonomic programming may increase their risk of developing hypertension later in life [21].

The present study shows that most hypertensive children were either obese (46.7%) or overweight (26.7%) and had lower HRV compared to normotensive children. Hypertension was associated with reduced parasympathetic activity (lower mean RR, SDNN, RMSSD, NN50, pNN50, and HFnu) and increased sympathetic activity (higher mean heart rate, LFnu, and stress index). The large effect sizes seen in geometric and stress-related HRV measures indicate clear sympathetic dominance and reduced overall autonomic balance in these children.

Kamińska et al. have explained that decreased parasympathetic drive with increased sympathetic tone might be related to hemodynamic transition from normotension to pre-hypertension, characterised by an elevated cardiac output and normal vascular resistance [22]. This high cardiac output has been associated with both an increased cardiac sympathetic drive and a decreased parasympathetic tone. In adults with hypertension, sympathetic nervous system activity is high in those with an impaired BP circadian rhythm, such as absent nocturnal dipping seen on ABPM [22]. Similarly, individuals with masked or white-coat hypertension also exhibit increased central sympathetic drive [22]. Urbina et al. performed a study in adolescents and suggested a shift toward sympathetic dominance with rising blood pressure [10]. Fitzgibbon et al. reported that children with higher BP exhibit reduced baroreflex sensitivity and decreased HRV [23]. Javorka et al. reported that during childhood, reduced parasympathetic activity is associated with cardiac autonomic neuropathy in the presence of elevated BP [24].

The significant positive association between BMI and LF/HF ratio in hypertensive children suggests a shift toward sympathetic dominance with increasing BMI. The absence of similar associations in normotensive children implies that hypertension may potentiate BMI-related autonomic imbalance. Overall, these findings indicate that relative autonomic indices such as LF/HF ratio may identify early autonomic imbalance before clear changes in HRV or blood pressure appear. Kaufman et al. also demonstrated that obese children exhibit cardiac autonomic dysfunction characterized by an increased LF/HF ratio [25]. Speer et al. suggested a link between autonomic function and BMI, showing that higher BMI was associated with lower cardiac vagal HRV in children aged three to five years [26]. Their findings suggest that autonomic activity is closely related to body composition and plays an important role in shaping cardiometabolic health even in early childhood [26].

Hirsch et al. showed that a 10% body weight gain significantly decreased HRV, which was attributable to decreased parasympathetic activity [27]. Increased weight and BMI are associated with high sympathetic and low parasympathetic indices. Masuo et al. have reported that plasma norepinephrine concentrations increase following weight gain in men [28]. Higher BP in obese children with isolated systolic hypertension is associated with increased heart rate and blood pressure variability (BPV) [29]. In obesity, excess visceral fat may activate central leptin-melanocortin pathways and immune inflammation, leading to chronic sympathetic overactivity and the development of hypertension in children [9].

In prehypertensive or early hypertensive children, a high LF/HF ratio may reflect an early disturbance in autonomic regulation that contributes to poor BP control and plays a role in the development of hypertension. In contrast, in chronic hypertension, a high LF/HF ratio is more often a consequence of sustained elevated BP, reflecting baroreflex dysfunction and cardiac autonomic neuropathy, rather than a healthy or balanced autonomic state [30].

Strengths and limitations

The use of ABPM to detect nocturnal and masked hypertension, along with simultaneous assessment of heart rate variability to explore underlying autonomic mechanisms, adds to the strength of our study. The integrated evaluation of BMI and autonomic indices further enhances understanding of obesity-related cardiovascular risk in children. However, the case-control design limits causal inference, and the relatively small sample size may affect generalizability. Future research should explore the role of leptin and its association with autonomic dysfunction and hypertension to better understand underlying pathophysiological mechanisms.

## Conclusions

Diagnosing hypertension and the factors that cause or worsen it at a young age can help prevent problems later in life. Our findings reaffirm the value of ABPM in revealing clinically silent nocturnal hypertension, a phenotype strongly linked to early target-organ damage. Hypertensive children demonstrated clear autonomic imbalance, characterised by sympathetic overactivity and parasympathetic withdrawal. Weight gain or obesity is hazardous, and factors like low physical activity and increased screen time contribute to overweight/obesity, which can raise sympathetic drive and trigger hypertension. Also, children with normal BMI may be at risk of developing hypertension due to other exacerbators. Early detection, lifestyle modification, and autonomic modulation strategies are essential to interrupt the trajectory from pediatric hypertension to lifelong cardiovascular disease.
